# Metabolic engineering of *Zymomonas mobilis* for anaerobic isobutanol production

**DOI:** 10.1186/s13068-020-1654-x

**Published:** 2020-01-25

**Authors:** Mengyue Qiu, Wei Shen, Xiongyin Yan, Qiaoning He, Dongbo Cai, Shouwen Chen, Hui Wei, Eric P. Knoshaug, Min Zhang, Michael E. Himmel, Shihui Yang

**Affiliations:** 1 State Key Laboratory of Biocatalysis and Enzyme Engineering, Environmental Microbial Technology Center of Hubei Province, and School of Life Sciences, Hubei University, Wuhan, 430062 China; 2 Biosciences Centers, National Renewable Energy Laboratory, Golden, CO 80401 USA; 3 National Bioenergy Center, National Renewable Energy Laboratory, Golden, CO 80401 USA

**Keywords:** *Zymomonas mobilis*, Biofuels, Isobutanol, Metabolic engineering, Pyruvate-derived biochemicals, 2-Ketoisovalerate decarboxylase (Kdc)

## Abstract

**Background:**

Biofuels and value-added biochemicals derived from renewable biomass via biochemical conversion have attracted considerable attention to meet global sustainable energy and environmental goals. Isobutanol is a four-carbon alcohol with many advantages that make it attractive as a fossil-fuel alternative. *Zymomonas mobilis* is a highly efficient, anaerobic, ethanologenic bacterium making it a promising industrial platform for use in a biorefinery.

**Results:**

In this study, the effect of isobutanol on *Z. mobilis* was investigated, and various isobutanol-producing recombinant strains were constructed. The results showed that the *Z. mobilis* parental strain was able to grow in the presence of isobutanol below 12 g/L while concentrations greater than 16 g/L inhibited cell growth. Integration of the heterologous gene encoding 2-ketoisovalerate decarboxylase such as *kdcA* from *Lactococcus lactis* is required for isobutanol production in *Z. mobilis*. Moreover, isobutanol production increased from nearly zero to 100–150 mg/L in recombinant strains containing the *kdcA* gene driven by the tetracycline-inducible promoter *Ptet*. In addition, we determined that overexpression of a heterologous *als* gene and two native genes (*ilvC* and *ilvD*) involved in valine metabolism in a recombinant *Z. mobilis* strain expressing *kdcA* can divert pyruvate from ethanol production to isobutanol biosynthesis. This engineering improved isobutanol production to above 1 g/L. Finally, recombinant strains containing both a synthetic operon, *als*-*ilvC*-*ilvD*, driven by *Ptet* and the *kdcA* gene driven by the constitutive strong promoter, *Pgap*, were determined to greatly enhance isobutanol production with a maximum titer about 4.0 g/L. Finally, isobutanol production was negatively affected by aeration with more isobutanol being produced in more poorly aerated flasks.

**Conclusions:**

This study demonstrated that overexpression of *kdcA* in combination with a synthetic heterologous operon, *als*-*ilvC*-*ilvD*, is crucial for diverting pyruvate from ethanol production for enhanced isobutanol biosynthesis. Moreover, this study also provides a strategy for harnessing the valine metabolic pathway for future production of other pyruvate-derived biochemicals in *Z. mobilis*.

## Background

Current global environment concerns provide motivation for the development of renewable and sustainable biofuels production technologies [1, 2]. In recent years, many microorganisms have been developed to produce eco-friendly, renewable biofuels using metabolic engineering and synthetic biology [3, 4]. Although bioethanol is the most studied bio-based fuel and lignocellulosic ethanol production has been successfully established in several countries, the intrinsic properties of bioethanol, such as high hygroscopicity, high vapor pressure, and low energy density limit its application in specific cases, such as jet fuel [5–7]. In contrast, higher alcohols, such as isobutanol, possess several advantages. These include higher energy density, low hygroscopicity, low vapor pressure, and high-octane number. Isobutanol can also serve as a precursor for the production of isobutene, making it a promising alternative for current fossil fuels [6, 8, 9]. In fact, Gevo Inc. has announced that it will develop and deploy isobutanol as a jet-fuel additive from renewable feedstocks (http://ir.gevo.com).

*Zymomonas mobilis* is a Gram-negative, natural bacterial ethanologen with many desirable industrial characteristics, such as a high specific rate of glucose uptake, low biomass production, low aeration cost due to anaerobic fermentation, and a high ethanol tolerance up to 16% (v/v) [2, 10–12]. *Z. mobilis* has been engineered to broaden its range of fermentable substrates, which now include the pentoses (xylose and arabinose) [13], and to expand its range of fermentation products to include 2,3-butanediol and lactic acid [1, 2, 4, 10, 14–16]. In addition, the genome sequence, functional re-annotation, and substantial systems biology studies and metabolic models of *Z. mobilis* have been reported [12, 15, 17–24]. Recent development of genome editing such as exogenous and native CRISPR-cas toolkits as well as methods for biological part identification and characterization [25–31] will facilitate heterologous pathway engineering and unravel the underlying mechanisms for balanced production and robustness [32] in *Z. mobilis*.

The isobutanol biosynthesis pathway involves five enzymes. First, acetolactate synthase (Als, EC2.2.1.6) catalyzes the condensation of two pyruvates to generate acetolactate, which is then reduced to 2,3-dihydroxyisovalerate by ketol-acid reductoisomerase (IlvC, EC1.1.1.86). This intermediate is subsequently converted to 2-ketoisovalerate by dihydroxy-acid dehydratase (IlvD, EC4.2.1.9), and further converted to isobutyraldehyde and alcohols (e.g., isobutanol) subsequently by 2-ketoisovalerate decarboxylase (Kdc) and alcohol dehydrogenase (Adh). Als usually contains a large subunit with catalytic function and a small (regulatory) subunit involved in substrate specificity, valine sensitivity and cofactor affinity, respectively. The first three enzymes are shared with the l-valine biosynthesis pathway, which exists in most microorganisms. Adh enzymes are also widespread in nearly all microorganisms. However, the essential enzyme, Kdc, for isobutanol production is absent in most bacteria including *Z. mobilis*. But some species of yeasts, fungi, and plants possess this enzyme [16], which can be introduced into microorganisms for heterologous isobutanol production.

The isobutanol biosynthesis pathway has been engineered into various microorganisms, such as *Escherichia coli* [33], *Saccharomyces cerevisiae* [7, 34], *Pichia pastoris* [35], *Bacillus subtilis* [36], *Corynebacterium glutamicum* [8, 37], *Geobacillus thermoglucosidasius* [38], and *Synechocystis* PCC 6803 [39–41]. For example, Atsumi et al. engineered *E. coli* to produce isobutanol up to 22 g/L in shake flasks [33, 42], which was further increased to 50 g/L in bioreactors during batch cultures with in situ product removal by gas stripping [43]. Recently, Ghosh and co-workers optimized their constructs based on models to predict the impacts of enzyme synthesis cost on cellular growth rates and obtained a recombinant strain with an isobutanol productivity of 3 g/h/g DCW (dry ell weight) [44]. Li and co-workers found that *B. subtilis* was a feasible host because it had a higher isobutanol tolerance than *E. coli* and *C. glutamicum* and produced up to 2.62 g/L isobutanol during fed-batch fermentation [36].

Atsumi and coworkers also introduced the isobutanol biosynthesis pathway genes from several species into the cyanobacterium *Synechococcus elongatus* PCC7942, confirming that the microorganism was able to produce an isobutanol titer of 450 mg/L directly from carbon dioxide [45]. Isobutanol production from *Synechocystis* PCC 6803 was also investigated and shown to achieve a cumulative titer of 911 mg/L following implementation of new metabolic engineering strategies [39, 40, 46].

As for eukaryotes, Chen and co-workers examined the feasibility of exploring *S. cerevisiae* as a potential platform microorganism for higher alcohols production. By overexpressing the *ilv2*, *ilv3*, and *ilv5* genes involved in valine metabolism, the authors were able to achieve an isobutanol yield of 3.86 mg/g glucose under aerobic conditions [7]. This isobutanol yield was further elevated to 6.6 mg/g glucose by overexpressing *kivd*, as well as *Adh6* and *ilv2* to enhance the endogenous enzyme activities necessary for isobutanol production. *pdc1* was also deleted to temper ethanol flux via pyruvate [34].

Despite of significant progress achieved on isobutanol production in various microorganisms, most require aerobic fermentation conditions, a costly input for industrial production. In addition, current efforts on improving isobutanol production in these microorganisms have been focused on increasing the pyruvate pool, eliminating by-product formation, and maintaining redox balance [44, 47–51]. For example, the Entner–Doudoroff (ED) pathway was applied into *E. coli*, and the ED pathway-dependent isobutanol-producing *E. coli* strain was further optimized to produce 15.0 g/L isobutanol after byproduct biosynthesis genes were inactivated [52]. *Z. mobilis* can be a suitable host to address these issues since it has the characteristics of a unique anaerobic ED pathway, a truncated tricarboxylic acid cycle (TCA), and simple metabolism resulting in abundant pyruvate biosynthesis with redox balancing that limits by-product formation. In this study, a heterologous isobutanol production pathway was engineered into a facultative anaerobic bacterium, *Z. mobilis*, for anaerobic isobutanol production, which will not only generate recombinant strains for anaerobic isobutanol production, but also provide strategies to divert pyruvate from the prevailing ethanol biosynthesis to the potential production of other pyruvate-derived biochemicals.

## Results and discussion

### Investigation of isobutanol toxicity to *Z. mobilis*

**Fig. 1:**
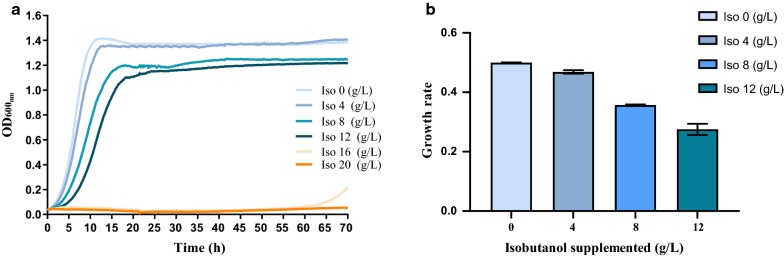
The impact of isobutanol concentration on the growth of *Z. mobilis*. The growth of *Z. mobilis* ZM4 in rich medium (RM) with isobutanol (Iso) supplementation at concentrations of 0, 4, 8, 12, 16, or 20 g/L (**a**), and the corresponding growth rates with 0, 4, 8, or 12 g/L isobutanol supplementation (**b**). At least two independent experiments have been carried out with similar results. Values are the mean of one representative experiment with three or more technical replicates. Error bars represent standard deviations

In order to produce potentially toxic biochemicals like isobutanol with high titer, yield, and productivity, the host microorganism must have the capability to tolerate the product at increased concentrations. Therefore, isobutanol toxicity to *Z. mobilis* was investigated. As shown in Fig. 1, *Z. mobilis* cannot grow in medium with 20 g/L isobutanol supplementation and had a lag phase of more than 60 h before showing growth in medium containing 16 g/L isobutanol. Although *Z. mobilis* can grow with the supplementation of isobutanol at 12 g/L or lower, both growth rates and final OD_600nm_ values were reduced correspondingly with the increase of supplemented isobutanol (Fig. 1). For example, compared to growth without isobutanol, the final OD_600nm_ value with the presence of 12 g/L isobutanol decreased only slightly from 1.4 to 1.2 (Fig. 1a). However, under these conditions, the growth rate dropped by nearly half from 0.50 ± 0.00 to 0.275 ± 0.019 (Fig. 1b). The reduction in growth rate and the corresponding increase in isobutanol concentration were correlated (*R*-squared value of 0.96; *y* = − 0.0196*x* + 0.5179) (Fig. 1b).

*Z. mobilis* can tolerate ethanol up to 16% (v/v) [1]. In our previous study, we characterized the toxicity of 2,3-butanediol on *Z. mobilis* and found that 2,3-butanediol was less toxic than ethanol and the supplementation of 100 g/L 2,3-butanediol only reduced growth rate by 20% [4]. These results indicate that although isobutanol is more toxic than ethanol and 2,3-butanediol to *Z. mobilis*, a titer up to 12 g/L could be achieved without completely preventing cells from growing, an outcome which can potentially be optimized for isobutanol production through tolerance engineering. Gas stripping during fermentation could also be applied to remove the isobutanol in situ to increase the titer, yield, and productivity. Ultimately, more robust strains could be developed using mutagenesis and adaptive laboratory evolution (ALE).

### Selection of genes for heterologous isobutanol production in *Z. mobilis*

**Fig. 2:**
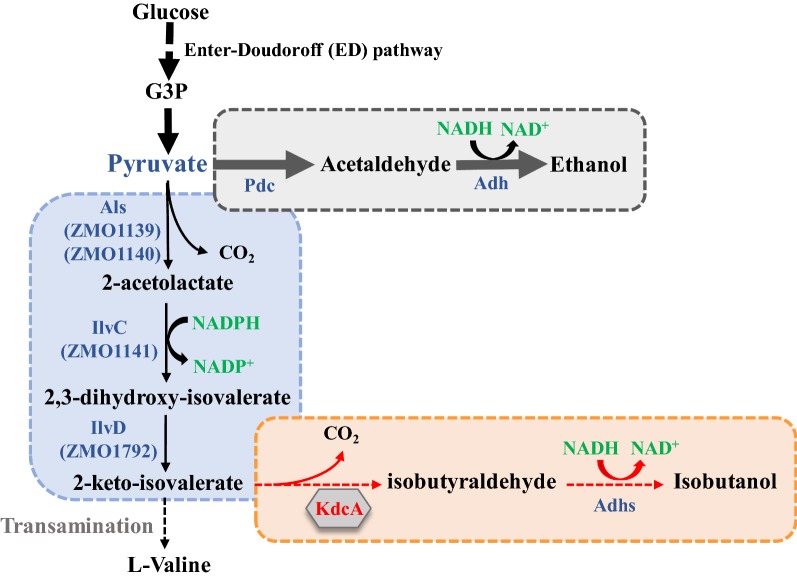
Isobutanol biosynthesis pathway and associated enzymes for heterologous isobutanol production in *Z. mobilis*. *G3P* glyceraldehyde 3-phosphate, *Als* acetolactate synthase, *IlvC* ketol-acid reductoisomerase, *IlvD* dihydroxy-acid dehydratase, *KdcA* 2-ketoacid decarboxylase, *Pdc* pyruvate dehydrogenase, *Adh* alcohol dehydrogenase, *Adhs* alcohol dehydrogenases. The codon-optimized heterologous KdcA from *Lactococcus lactis* for isobutanol production has red-color font in the gray-color shaded diamond shape. Enzymes with blue-color font refer to native genes in *Z. mobilis*

Based on gene homology search results, *Z. mobilis* possesses four genes ZMO01139, ZMO1140, ZMO1141, and ZMO1792 encoding the enzymes Als, IlvC, and IlvD, respectively. ZMO01139 and ZMO1140 encode the large and small (regulatory) subunits of Als, respectively. These enzymes are the first three enzymes in the production of l-valine from pyruvate (Fig. 2). In addition, several alcohol dehydrogenases were also identified in *Z. mobilis* including a Zn-dependent alcohol dehydrogenase AdhA (ZMO1236); iron-containing alcohol dehydrogenases AdhB (ZMO1593) and ZMO1771; a Zn-dependent alcohol dehydrogenase class III AdhC (ZMO1722); and a plasmid-borne alcohol dehydrogenase-like protein (pZYM33_021).

Our previous study demonstrated that one of these five alcohol dehydrogenases contributes to 2,3-butanediol production [4], which may also be functional for the conversion of isobutyraldehyde to isobutanol although further study is needed to determine the roles of these Adh enzymes in the biosynthesis of different alcohols. The only enzyme required for isobutanol biosynthesis that was not identified in *Z. mobilis* is *kdcA*. The *kdcA* gene from *Lactococcus lactis* was used in previous studies for heterologous isobutanol production, which was selected and the codon-optimized *kdcA* gene was synthesized by GenScript (Nanjing, China) for subsequent study.

### Introduction of *kdcA* into *Z. mobilis* for isobutanol production

The codon-optimized *kdcA* gene from *L. lactis* was cloned into the shuttle vector pEZ15Asp [4] generating the plasmid pEZ-KT, in which *kdcA* is driven by the tetracycline-inducible promoter *Ptet*. pEZ-KT was then introduced into *Z. mobilis* ZM4 to generate the recombinant strain ZM4-KT, and then confirmed by Sanger sequencing.

**Fig. 3:**
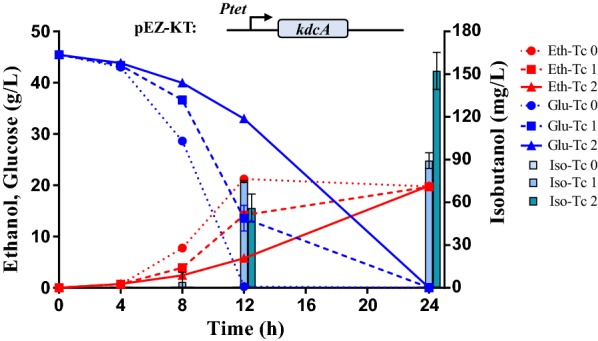
Glucose (Glu) consumption, isobutanol (Iso) and ethanol (Eth) production of *Z. mobilis* strain ZM4-KT in RMG5 with different concentrations of tetracycline. The codon-optimized *kdcA* gene from *L. lactis* driven by the *Ptet* inducible promoter was cloned into the shuttle vector pEZ15Asp, which was then confirmed and transformed into *Z. mobilis* strain ZM4 to generate the recombinant strain ZM4-KT. Tc 0, Tc 1 and Tc 2 represented the tetracycline concentrations of 0, 0.2, and 1.0 μg/mL that was added into the medium one time at the beginning of the experiment, respectively. At least two independent experiments were carried out with similar results. Values are the mean of one representative experiment with three technical replicates. Error bars represent standard deviations

The impact of *kdcA* expression under the control of the *Ptet* promoter on isobutanol production was then investigated with different concentrations of tetracycline as an inducer ranging from 0 to 2 μg/mL. Isobutanol production was significantly improved with the increase of tetracycline concentrations (Fig. 3). When glucose was completely consumed (i.e., within 24 h of post-inoculation), the isobutanol titer was 89.33 ± 5.51 mg/L and 152.33 ± 13.05 mg/L with the induction of 1 and 2 μg/mL tetracycline, respectively. No isobutanol was detected without tetracycline induction (Fig. 3).

Although there were no significant differences in final ethanol titers (around 20 g/L) when induced by different concentrations of tetracycline, glucose consumption and ethanol production rates decreased with the increase of tetracycline concentrations (Fig. 3). Our previous study indicated that tetracycline negatively affected cellular growth of *Z. mobilis* above 2 μg/mL. However, tetracycline almost had no impact at concentrations below 1 μg/mL [28]. Considering the titers of isobutanol were around 40–150 mg/L level, the decreased glucose utilization and ethanol production rates may be due to the impact of tetracycline on cellular function at high concentrations (i.e., 2 μg/mL). Tetracycline concentrations of 0, 0.2, and 1 μg/mL were therefore selected for subsequent experiments.

### Construction of stable heterologous isobutanol production strains

**Fig. 4:**
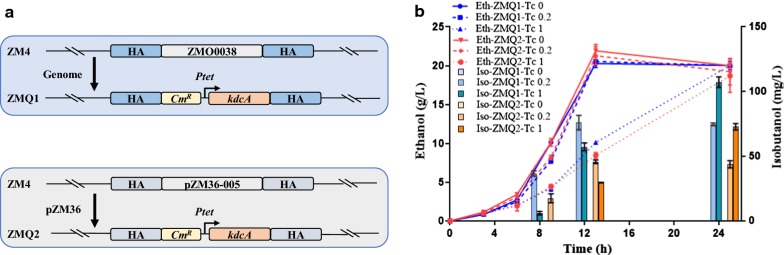
Integration of heterologous *KdcA* gene from *L. lactis* into the chromosome or a native plasmid of *Z. mobilis* for stable isobutanol production. ZMQ1 and ZMQ2 were constructed by integrating the *kdc*A gene driven by tetracycline-inducible promoter *Ptet* into the ZMO0038 chromosomal locus or the pZM36-005 locus in the native plasmid pZM36, respectively (**a**). Concentrations of isobutanol (Iso) and ethanol (Eth) for *Z. mobilis* strains ZMQ1 and ZMQ2 in RMG5 with tetracycline induction at 0, 0.2, and 1 μg/mL (**b**). Tc 0, Tc 0.2 and Tc 1 represented the tetracycline concentrations of 0, 0.2, and 1.0 μg/mL that was added into the medium one time at the beginning of the experiment, respectively. At least two independent experiments were carried out with similar results. Values are the mean of one representative experiment with three technical replicates. Error bars represent standard deviations

*Zymomonas mobilis* ZM4 contains a 2.06 Mb genome with four native plasmids (pZM32 (32,791 bp), pZM33 (33,006 bp), pZM36 (36,494 bp), and pZM39 (39,266 bp) [15]. ZMO0038 is a putative sigma 54 modulation protein and the deletion of ZMO0038 does not affect the growth of *Z. mobilis* [53]. To construct a stable isobutanol production strain, the codon-optimized *kdcA* gene driven by *Ptet* was integrated into the genome at either the chromosomal locus of ZMO0038 or the native plasmid locus pZM36-005, resulting in the recombinant strains of ZMQ1 and ZMQ2, respectively (Fig. 4a). Tetracycline concentrations of 0, 0.2, and 1 μg/mL were used to induce the expression of *kdcA* for isobutanol production. ZMQ1 reached a maximum isobutanol titer of 104.33 ± 3.51 mg/L, which was 1.4 times higher than ZMQ2 with a titer of 74.67 ± 0.58 mg/L with 1 μg/mL tetracycline induction 24-h post-inoculation (Fig. 4b). Since the expression of the *kdcA* gene in the ZM4 genome at the ZMO0038 locus was better than in the native plasmid pZM36, ZMQ1 was selected for further experiments.

In addition, although the titer of isobutanol increased corresponding to the higher tetracycline concentrations, the final ethanol titer was nearly the same between ZMQ1 and ZMQ2 with both reaching approximately 20 g/L. These results indicated that the *kdcA* gene alone cannot effectively compete with ethanol production and another strategy is needed to divert carbon flux from ethanol production to isobutanol biosynthesis.

### Overexpression of heterologous *als* and *ilvC*-*ilvD* to increase isobutanol production

**Fig. 5:**
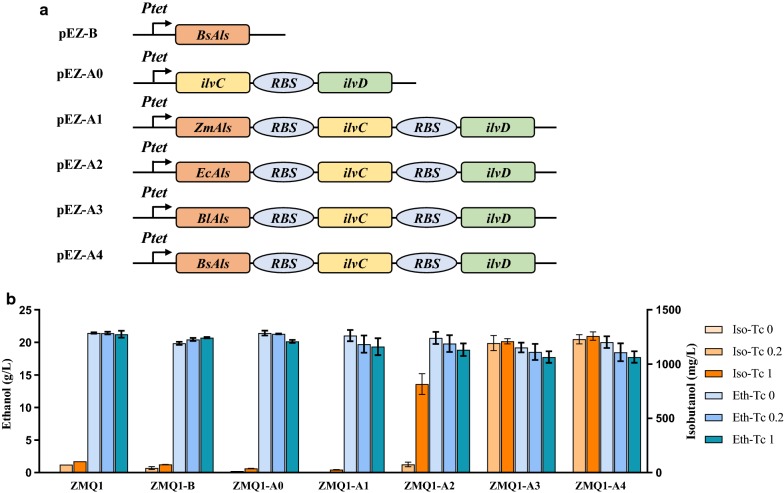
Plasmid constructs to divert carbon flux from ethanol production to isobutanol biosynthesis and their performance in recombinant strains. Six plasmids (pEZ-B, pEZ-A0, pEZ-A1, pEZ-A2, pEZ-A3, and pEZ-A4) containing different combinations of four *als* genes from different bacteria (*ZmAls* from *Z. mobilis*, *EcAls* from *E. cloacae*, *BlAls* from *Bacillus licheniformis*, and *BsAls* from *B. subtilis*) as well as *ilvC* and *ilvD* genes from *Z. mobilis* for isobutanol production (**a**). Ethanol (Eth) and isobutanol (Iso) production in the recombinant *Z. mobilis* strains ZMQ1-B, ZMQ1-A0, ZMQ1-A1, ZMQ1-A2, ZMQ1-A3, and ZMQ1-A4 after 24 h post-induction in RMG5 with different tetracycline inducer concentrations (**b**). Tc 0, Tc 0.2 and Tc 1 represented the tetracycline concentrations of 0, 0.2, and 1.0 μg/mL that was added into the medium one time at the beginning of the experiment, respectively. At least two independent experiments were carried out with similar results. Values are the mean of one representative experiment with three or more technical replicates. Error bars represent standard deviations

Our previous work indicated that the addition of a heterologous Als is crucial to divert carbon flux from ethanol production to 2,3-butanediol biosynthesis [4]. To enhance isobutanol production, a similar strategy was employed. Four *als* genes from different bacteria (*ZmAls* from *Z. mobilis*, *EcAls* from *E. cloacae*, *BlAls* from *Bacillus licheniformis*, and *BsAls* from *B. subtilis*) as well as *ilvC* and *ilvD* from *Z. mobilis* were selected and six plasmids were constructed (Fig. 5a). Since the *als* gene is driven by a strong promoter, *Pgap*, and may cause increased cellular metabolic burden consistent with our previous observations [4], an inducible promoter *Ptet* was utilized to control the expression of the synthetic operon containing the *als*, *ilvC,* and *ilvD* genes (Fig. 5a). We then examined the impacts on *Z. mobilis* isobutanol production by inducing Als alone, both IlvC and IlvD, and combinations of Als from four other bacteria. The IlvC and IlvD constructs contain a strong bacterial ribosomal binding site (RBS) (BBa_B0034) (Fig. 5b) are detailed below [28].

Isobutanol production in the recombinant strain, ZMQ1-B, containing the *als* gene from *B. subtilis* decreased to 75.33 ± 0.58 mg/L compared to 104.33 ± 3.51 mg/L in the host strain ZMQ1. Isobutanol production in the recombinant strains ZMQ1-A0 and ZMQ1-A1 containing plasmids pEZ-A0 and pEZ-A1 to overexpress *ilvC* and *ilvD* or all three genes *ZmAls*, *ilvC*, and *ilvD* also decreased to 38.33 ± 1.15 mg/L and 26.67 ± 2.08 mg/L, respectively, under the induction of 1 μg/mL tetracycline (Fig. 5b). These results suggest that the overexpression of only *BsAls* or *ilvC* and *ilvD*, or all three native genes *ZmAls*, *ilvC*, and *ilvD*, were not able to increase isobutanol production.

However, the overexpression of a heterologous operon containing an *als* gene (*EcAls*, *BlAls*, or *BsAls*) with both *ilvC* and *ilvD* in ZMQ1-A2, ZMQ1-A3, and ZMQ1-A4 increased isobutanol production to 0.82 ± 0.09 g/L, 1.05 ± 0.12 g/L, and 1.23 ± 0.05 g/L with the induction of 1 μg/mL tetracycline (Fig. 5b), which was about 8, 12, and 12.6 times of the isobutanol production in ZMQ1, respectively. Correlated with the increase of isobutanol production in ZMQ1-A2, ZMQ1-A3, and ZMQ1-A4, ethanol production decreased from 21.44 ± 0.11 g/L in ZMQ1 to 18.86 ± 0.96, 17.74 ± 0.89, and 17.75 ± 0.87 g/L in these recombinant strains, respectively (Fig. 5b). Ethanol production in the recombinant strains ZMQ1-A2, ZMQ1-A3, and ZMQ1-A4 without tetracycline induction was comparable to ZMQ1, with a titer of 20.67 ± 0.93, 19.21 ± 0.75, 20.03 ± 0.89, respectively. These results indicate that in *Z. mobilis*, a non-native *als* gene coupled with over-expression of the native *ilvC*-*ilvD* genes are necessary to divert carbon flux from ethanol production or native valine biosynthesis to isobutanol production.

### Replacement of the inducible *Ptet* promoter driving *kdcA* with constitutive strong promoters to increase isobutanol production

The *Ptet* promoter was used in this study to evaluate the effects of overexpression of either the *kdcA* gene or the synthetic operon of *als*-*ilvC*-*ilvD* on isobutanol production. Our results demonstrated that by increasing the concentrations of the tetracycline inducer, isobutanol titers could also be increased (Figs. 3, 4, 5).

Our previous study on constructing the 2,3-butanediol production strains demonstrated that the expression of a synthetic operon *aldC*-*bdh* under the control of the constitutive strong promoter *Pgap* is crucial for high 2,3-BDO production, and the combination of *Ptet* driving the heterologous *als* gene with the *Pgap* driving *aldC*-*bdh* gave the maximum 2,3-BDO production with a titer of 13.3 ± 0.7 g/L [4]. We therefore explored the possibility of increasing isobutanol production by replacing the *Ptet* promoter driving the *kdcA* gene with a strong promoter of *Peno* or *Pgap* while keeping *Ptet* to drive the expression of the *als*-*ilvC*-*ilvD* operon.

**Fig. 6:**
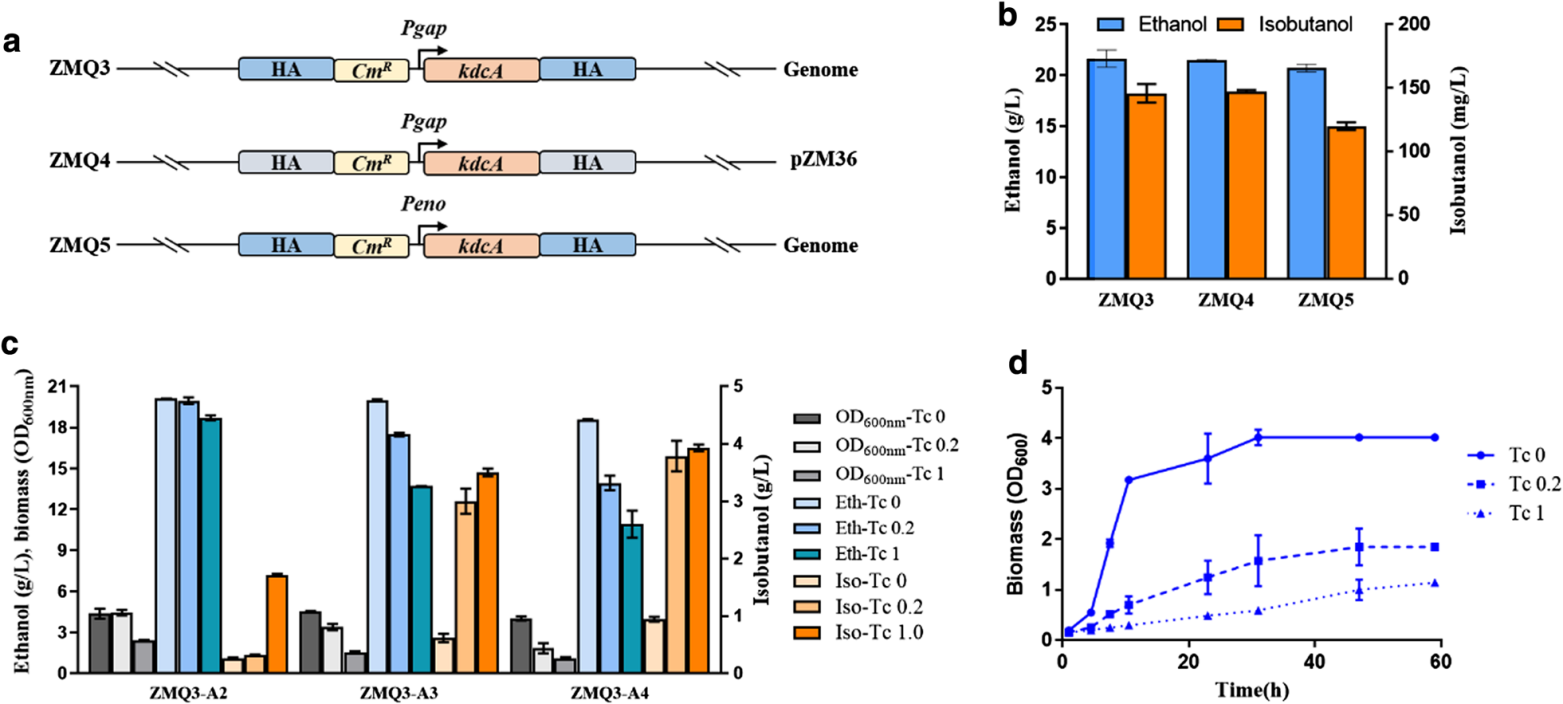
Strategy to improve isobutanol production in *Z. mobilis* by replacing the inducible *Ptet* promoter driving the *kdcA* gene with different constitutive strong promoters. *Ptet* promoter was replaced with three recombinant strains constructed for isobutanol production. ZMQ3, *kdcA* driven by *Pgap* was integrated at chromosomal gene ZMO0038 locus; ZMQ4, *kdcA* driven by *Pgap* was integrated at the native plasmid pZM36-005 locus; ZMQ5, *kdcA* driven by *Peno* was integrated at chromosomal gene ZMO0038 locus (**a**). Ethanol and isobutanol production of *Z. mobilis* strains ZMQ3, ZMQ4, and ZMQ5 12-h post-inoculation in RMG5 (**b**). Growth, as well as ethanol (Eth) and isobutanol (Iso) production in ZMQ3-A2, ZMQ3-A3, and ZMQ3-A4 in RMG5 with the tetracycline induction at different concentrations (**c**). Growth curve of ZMQ3-A4 in RMG5 under the induction of different tetracycline concentrations of 0, 0.2, and 1 μg/mL (**d**). Tc 0, Tc 0.2 and Tc 1 represented the tetracycline concentrations of 0, 0.2, and 1.0 μg/mL that was added into the medium one time at the beginning of the experiment, respectively. At least two independent experiments were carried out with similar results. Values are the mean of one representative experiment with three or more technical replicates. Error bars represent standard deviations

First, we replaced the *Ptet* promoter in ZMQ1 and ZMQ2 with the strong promoter *Pgap* and generated the new recombinant strains ZMQ3 (integration at the ZMO0038 chromosomal locus) and ZMQ4 (integration at the pZM36-005 native plasmid locus), respectively. In addition, the *Ptet* promoter was also replaced with another constitutive strong promoter, *Peno,* with integration at the ZMO0038 chromosomal locus to generate ZMQ 5 (Fig. 6a). The titers of isobutanol production in ZMQ3, ZMQ4, and ZMQ5 were 144.33 ± 7.23, 147.33 ± 1.15, and 120.00 ± 3.00 mg/L under the induction of 1 μg/mL tetracycline 12 h post-inoculation, respectively (Fig. 6b). The titers and productivities of ZMQ3, ZMQ4, and ZMQ5 were all significantly higher than those of ZMQ1 and ZMQ2, which were 104.33 ± 3.51 and 74.67 ± 0.58 mg/L after 24 h inoculation, respectively. Because isobutanol production in ZMQ3 and ZMQ4 were comparable to each other, but higher than that in ZMQ5, ZMQ3 with the *kdcA* gene driven by the constitutive strong promoter, *Pgap,* inserted into the ZMO0038 locus was selected for subsequent experiments.

Three plasmids (pEZ-A2, pEZ-A3, and pEZ-A4) with *Ptet* driving the expression of the synthetic operon, *als*-*ilvC*-*ilvD* (Fig. 5a), were then transformed into the recombinant ZMQ3 separately to generate the recombinant strains ZMQ3-A2, ZMQ3-A3, and ZMQ3-A4, respectively. Isobutanol production in these three recombinants was significantly higher than the parental strain ZMQ3 with the maximum isobutanol production in ZMQ3-A4 reaching 4.01 ± 0.06 g/L. The overexpression of the heterologous *BsAls* gene from *B. subtilis* was the most effective way to divert carbon flux into isobutanol biosynthesis. Concurrently, recombinant strains showed diminishing ethanol production with increasing tetracycline concentrations used for induction and isobutanol production (Fig. 6c). For instance, the isobutanol production of ZMQ3-A4 reached a titer of 4.01 ± 0.06 g/L, which was four times of that without tetracycline induction, while ethanol production in ZMQ3-A4 decreased to 10.93 ± 0.99 g/L, 50% of ZMQ3-A4 without tetracycline induction (Fig. 6c). Therefore, our results indicated that the overexpression of the synthetic operon, *als*-*ilvC*-*ilvD* and *kdcA*, could enhance isobutanol production by diverting carbon flux from ethanol biosynthesis to isobutanol biosynthesis pathway.

Growth of the recombinant strain ZMQ3-A4 with the highest isobutanol titer was further examined in this study. The results showed that although isobutanol titer increased gradually, the growth rates of ZMQ3-A4 dropped with the increase of tetracycline inducer concentrations (Fig. 6d). As discussed above, the growth rate of *Z. mobilis* with the supplementation of 4 g/L isobutanol (0.47 ± 0.01) was almost the same as that without isobutanol supplementation (0.50 ± 0.00). Thus, the maximum isobutanol titer produced in this study by ZMQ3-A4 is not likely to affect cellular growth significantly. Together with the fact that the tetracycline concentrations used to induce the synthetic operon *als*-*ilvC*-*ilvD* does not affect the normal growth of *Z. mobilis*, slight growth inhibition in the ZMQ3-A4 recombinant strain under these highly inducing tetracycline concentrations is likely due to the protein production burden, precursor competition, and electron balancing resulting from the increased expression of isobutanol biosynthesis pathway genes, similar to that observed in a 2,3-butanediol production recombinant strain previously reported [4].

Although the recombinant strain ZMQ3-A4 had the highest isobutanol titers among three recombinant strains ZMQ3-A2, ZMQ3-A3, and ZMQ3-A4 under each tetracycline induction conditions (Fig. 6C), ZMQ3-A4 had higher isobutanol yield (%) with the tetracycline induction at concentrations of 0.2 and 1.0 μg/mL than those of ZMQ3-A2 and ZMQ3-A3 (Additional file 1: Table S1). The difference between ZMQ3-A3 and ZMQ3-A4 is *als* gene while ZMQ3-A3 used *als* gene from *B. licheniformis* and ZMQ3-A4 uses *als* gene from *B. subtilis* (Fig. 5a). The impact of heterologous pathway and different heterologous pathway genes on endogenous metabolic pathways and regulatory networks as well as the compatibility between heterologous pathways and chassis cells should also be investigated to help improve the titer, yield, and productivity of isobutanol production.

In addition, although our study gradually increased the isobutanol production through metabolic engineering with a titer up to 4.01 g/L, which is equal to or higher than the titer using other model species such as yeast *S. cerevisiae* (2.09 g/L), *B. subtilis* (2.6 g/L)*, Ralstonia eutropha* (0.85 g/L), and *S. elongatus* (0.45 g/L), it is still far less than the titers achieved using *E. coli* (50 g/L) and *C. glutamicum* (72.69 g/L) [50, 54]. Significant effort is still needed to further improve isobutanol tolerance and increase isobutanol titer, yield, and productivity including in situ product removal by gas stripping, knockout of pyruvate decarboxylase (Pdc) to redirect carbon flux from ethanol production into isobutanol production, as well as mutagenesis strategies such as ALE and atmospheric and room temperature plasma (ARTP) mutagenesis [55, 56].

### The effect of oxygen on isobutanol production

**Fig. 7:**
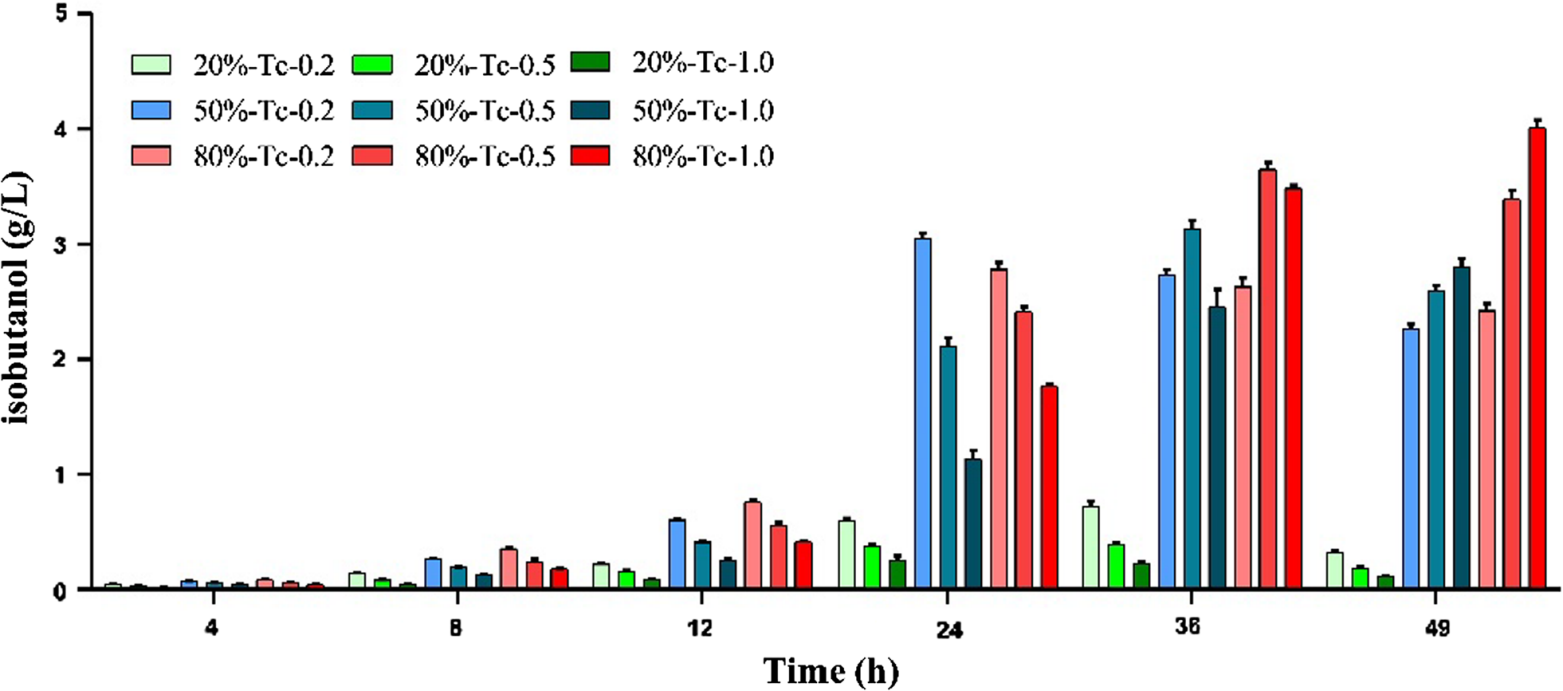
Isobutanol production of *Z. mobilis* recombinant strain ZMQ3-A4 in flask with different medium volumes of RMG5 medium (20, 50, or 80% flask volume) under the induction of different tetracycline concentrations of 0.2, 0.5 and 1.0 μg/mL, respectively. Tc 0, Tc 0.2, and Tc 1 represented the tetracycline concentrations of 0, 0.2, and 1.0 μg/mL that was added into the medium one time at the beginning of the experiment, respectively. The results shown are the mean of two technical replicates and the error bars represent standard deviations

As discussed above, although significant progress was made for isobutanol production through metabolic engineering in different microorganisms, nearly all of them are aerobic microorganisms producing isobutanol under aerobic conditions even though *E. coli* and *S. cerevisiae* were recently engineered for anaerobic isobutanol production [7, 57]. Since *Z. mobilis* is a facultative anaerobic ethanologen, we investigated the impact of the amount of oxygen on isobutanol production using shake flasks containing different medium volumes of 20, 50, or 80% of the capacity of the flask similar to our previous work for 2,3-butanediol production [4]. Our results showed that isobutanol production was affected by the oxygenation of the medium in the shake flask. More isobutanol was produced in shake flasks with a higher medium volume which contains less oxygen due to poorer mixing (Fig. 7).

Correlated with the low isobutanol production in the 20% volume condition in shake flask fermentation, glucose consumption and culture growth were impeded with less than 4.22 g/L ethanol produced after consuming almost all of the glucose (Additional file 2: Fig. S1A, Additional file 3: Table S2). In the 50% and 80% medium volume conditions, there was no significant change in cellular growth. However, when same concentration of tetracycline inducer was used, the titers of both ethanol and isobutanol under 80% medium volume condition were more than those under 50% medium volume condition (Additional file 2: Fig. S1B, C; Additional file 3: Table S2).

Concurrent with the reduced glucose utilization and cellular growth in the 20% medium volume condition, the byproducts of glycerol, acetate, and acetoin were increased (Additional file 2: Fig. S1D, Additional file 3: Table S2) consistent with our previous study for *Z. mobilis* in aerobic and anaerobic conditions [12]. Moreover, when the same concentrations of tetracycline were used, the productivity of almost all byproducts in the 20% medium volume condition were decreased compared to those in the 80% medium volume condition (Additional file 2: Fig. S1D–F; Additional file 3: Table S2). With the increase of fermentation medium volume, less byproducts will be produced (Additional file 2: Fig. S1D–F; Additional file 3: Table S2), which again is consistent with the results of our previous study [12]. All these results suggested that fermentation in an anoxic condition is a better choice for isobutanol production in *Z. mobilis.*

A well-balanced, low NADH/NAD^+^ ratio is the key for efficient glycolysis and cellular growth in *Z. mobilis*. Our previous study indicated that growth conditions impacting oxygen dispersion such as shaking speed and medium volume affected acetoin and 2,3-BDO production, and a microaerophilic condition best suitable for 2,3-BDO production [4]. Furthermore, the decrease of oxygen availability with the increase of flask medium volume can help sustain a more anoxic condition for efficient ethanol fermentation, which in return will produce more CO_2_ to retain the anoxic condition during its active exponential growth phase. On the contrary, the decrease of medium volume in the flask will result in the increase of amount of oxygen, and the competition between oxygen respiration and fermentation. To help maintain the redox balance due to the NADH oxidization under aerobic condition, more byproducts of acetoin, acetate, lactate, and glycerol might therefore be produced resulting in less ethanol and/or isobutanol production as reported in the literature [4, 12], which is same as what we observed in this study (Additional file 2: Fig. S1; Additional file 3: Table S2).

Similar to heterologous 2,3-BDO production, only one NAD^+^ will be regenerated from isobutanol biosynthesis compared to two NAD^+^ from ethanol fermentation. The reduced NAD^+^ generated via the heterologous isobutanol or 2,3-BDO metabolic pathway and the competition for substrate towards the production of byproducts such as glycerol may help increase the NADH/NAD^+^ ratio to maintain a balanced redox [4]. However, the requirement of oxygen for efficient isobutanol production with highest isobutanol titer achieved under fermentation in an anoxic condition (Fig. 7) was different from that for heterologous 2,3-butanediol (BDO) production in *Z. mobilis,* where microaerophilic condition was most suitable for 2,3-BDO production [4]. Therefore, further investigation is needed to understand the relationship among oxygen availability, growth phase, respiratory chain, NADH/NAD^+^ ratio, native glycolysis, and ethanol fermentation pathways for efficient isobutanol biosynthesis in *Z. mobilis*.

## Conclusion

In summary, the effect of exogenous isobutanol on *Z. mobilis* was investigated and various recombinant strains for isobutanol production were constructed. *Z. mobilis* can grow with the supplementation of isobutanol up to 12 g/L, and the integration of a heterologous 2-ketoisovalerate decarboxylase gene (*kdcA*) is required for isobutanol production in *Z. mobilis*. In addition, recombinant strains containing a *kdcA* gene driven by the constitutive strong promoter *Pgap* (*Pgap*-*kdcA*), as well as a synthetic operon *Ptet*-*als*-*ilvC*-*ilvD* containing a heterologous *als* and native *ilvC* and *ilvD* genes driven by *Ptet* were shown to be able to divert carbon flux from ethanol production into isobutanol biosynthesis. These recombinant strains produced a maximum isobutanol titer up to 4.01 g/L accompanied by a corresponding decrease in ethanol production. This work also provides a strategy for harnessing a heterologous valine metabolic pathway for the production of other pyruvate-derived biochemicals in *Z. mobilis*.

## Methods

### Strains, vectors, and media

The plasmids and strains used in this study are listed in Tables 1 and 2, respectively. *E. coli* DH5α was used for plasmid construction and *E. coli* Trans110 (*rpsL (Str*^*R*^*) thr leu thi*-*1 lacY galK galT ara tonA tsx dam dcm supE44 Δ(lac*-*proAB)/F′ [traD36proAB lacIq lacZΔM15]*) was used as the host for plasmid demethylation. *Z. mobilis* ZM4 was the parent strain for genetic modifications. All *E. coli* strains were cultured in Luria–Bertani medium (LB, 10 g/L tryptone, 5 g/L yeast extract, 10 g/L NaCl) at 37 °C and *Z. mobilis* strains were cultured in rich medium (RM, 10 g/L yeast extract, 45 g/L glucose, 2 g/L KH_2_PO_4_) at 30 °C. When required, antibiotics were used at the following final concentrations: spectinomycin (100 μg/mL) and chloramphenicol (50 μg/mL).

**Table 1:** Plasmids used in the study

Plasmids	Description	Source
pEZ15Asp (pEZ)	Shuttle vector contains *Z. mobilis* origin and *E. coli* origin p15A; *Sp*^*R*^;Biobrick-compatible	Lab stock
pEZ-KT	*Sp*^*R*^; pEZ containing: *kdcA* gene from *L. lactic* driven by inducible promoter *Ptet*	This work
pEZ-A0	*Sp*^*R*^; pEZ containing; *ilvC* and *ilvD* genes from *Z. mobilis* driven by inducible promoter *Ptet*	This work
pEZ-A1	*Sp*^*R*^; pEZ containing: *als*, *ilvC* and *ilvD* genes from *Z. mobilis* driven by inducible promoter *Ptet*	This work
pEZ-A2	*Sp*^*R*^; pEZ containing: codon-optimized *EcAls* gene from *E. cloacae*, *ilvC* and *ilvD* genes from *Z. mobilis* driven by *Ptet*	This work
pEZ-A3	*Sp*^*R*^; pEZ containing: codon-optimized *BlAls* from *B. licheniformis*, *ilvC* and *ilvD* from *Z. mobilis* driven by *Ptet*	This work
pEZ-A4	*Sp*^*R*^; pEZ containing: codon-optimized *BsAls* gene from *B. subtilis*, *ilvC* and *ilvD* genes from *Z. mobilis* driven by *Ptet*	This work
pEZ-B	*Sp*^*R*^; pEZ containing: codon-optimized *BsAls* gene from *B. subtilis* driven by inducible promoter *Ptet*	
Plasmids for recombination		
pUC57	Vector contains *E. coli* origin; Amp^R^	
pUC-KT1	*Amp*^*R*^*, Cm*^*R*^; pUC containing: chloramphenicol resistant gene, *kdcA* gene from *L. lactic* driven by *Ptet* and the upstream and downstream of ZMO0038	This work
pUC-KT2	*Amp*^*R*^*, Cm*^*R*^; pUC containing: chloramphenicol resistant gene, *kdcA* gene from *L. lactic* driven by *Ptet* and the upstream and downstream of pZYM36-005	This work
pUC-KG1	*Amp*^*R*^*, Cm*^*R*^; pUC containing: chloramphenicol resistant gene, *kdcA* gene from *L. lactic* driven by *Pgap* and the upstream and downstream of ZMO0038	This work
pUC-KG2	*Amp*^*R*^*, Cm*^*R*^; pUC containing: chloramphenicol resistant gene, *kdcA* gene from *L. lactic* driven by *Pgap* and the upstream and downstream of pZYM36-005	This work
pUC-KE	*Amp*^*R*^*, Cm*^*R*^; pUC containing: chloramphenicol resistant gene, *kdcA* gene from *L. lactic* driven by *Peno* and the upstream and downstream of ZMO0038	This work

**Table 2:** Strains used in the study

Strains	Description	Source
DH5α	*E. coli* for plasmid construction	Lab stock
Trans110	*E. coli* for plasmid demethylation	Lab stock
ZM4	*Zymomonas mobilis* subsp. *mobilis* ZM4 strain	Lab stock
ZM4-KT	ZM4 containing plasmid pEZ-KT	This work
ZMQ1	*Cm*^*R*^; recombination through plasmid pUC-KT1	This work
ZMQ1-B	*Sp*^*R*^, *Cm*^*R*^; ZMQ1 containing plasmid pEZ-B	This work
ZMQ1-A0	*Sp*^*R*^, *Cm*^*R*^; ZMQ1 containing plasmid pEZ-A0	This work
ZMQ1-A1	*Sp*^*R*^, *Cm*^*R*^; ZMQ1 containing plasmid pEZ-A1	This work
ZMQ1-A2	*Sp*^*R*^, *Cm*^*R*^; ZMQ1 containing plasmid pEZ-A2	This work
ZMQ1-A3	*Sp*^*R*^, *Cm*^*R*^; ZMQ1 containing plasmid pEZ-A3	This work
ZMQ1-A4	*Sp*^*R*^, *Cm*^*R*^; ZMQ1 containing plasmid pEZ-A4	This work
ZMQ2	*Cm*^*R*^; recombination through plasmid pUC-KT2	This work
ZMQ3	*Cm*^*R*^; recombination through plasmid pUC-KG1	This work
ZMQ3-A2	*Sp*^*R*^*, Cm*^*R*^; ZMQ3 containing plasmid pEZ-A2	This work
ZMQ3-A3	*Sp*^*R*^*, Cm*^*R*^; ZMQ3 containing plasmid pEZ-A3	This work
ZMQ3-A4	*Sp*^*R*^*, Cm*^*R*^; ZMQ3 containing plasmid pEZ-A4	This work
ZMQ4	*Cm*^*R*^; recombination through plasmid pUC-KG2	This work
ZMQ5	*Cm*^*R*^; recombination through plasmid pUC-KE	This work

### DNA manipulation techniques

For all plasmid constructions, the steps are as follows. Primers were designed to contain 15–20 nucleotides (nts) overlapping regions with adjacent DNA fragments. PCR products amplified by primer pairs were separated by gel electrophoresis, followed by column purification, and subsequently quantified using a NanoDrop spectrophotometer (NanoDrop Technologies, Wilmington, DE, USA). Fragments and vectors were mixed in a molar ratio of 3:1, 0.5 U T5 exonuclease (NEB, USA), and 0.5 μL buffer 4 (NEB, USA) were added in a final volume of 5 μL. All regents were mixed and incubated on ice for 5 min. The DNA reaction was added to chemically competent *E. coli* cells, incubated on ice for 30 min, heat shocked for 45 s at 42 °C, and held on ice for 2 min. Subsequently, 100 μL of NZY medium (yeast extract 5.0 g/L, NaCl 5 g/L, MgCl_2_ 1.2 g/L, MgSO_4_ 1.5 g/L, glucose 3.6 g/L, casein enzymatic hydrolysate NZ amine^®^ 10 g/L), was added to the mixture and recovered for at least 1 h at 37 °C with shaking (250 rpm).

Cells were plated on LB agar plates containing appropriate antibiotics. Recombinants were selected by colony PCR and confirmed by Sanger sequencing (TsingKe Biological Technology, Wuhan, China). The universal primers 15A-fwd/rev or M13-fwd/rev was used for colony PCR to check the insert size. Colonies containing correct plasmids with expected PCR product length through colony PCR screenings were cultivated in LB medium containing appropriate antibiotics for plasmid extraction, which was further Sanger sequencing confirmed (Tsingke Biological Technology, Wuhan, China).

Genetic modification of *Z. mobilis* was based on homologous recombination using a pUC57 suicide vector, which is unstable due to the lack of *Z. mobilis* replicative elements. The gene expression cassette flanking 1-kb upstream and 1-kb downstream of editing sites was assembled into the pUC57 vector. The customized specific plasmid was then transferred into *Z. mobilis* competent cells. Gene insertions were screened by colony PCR with the primer pair upstream-F and downstream-R, and further confirmed by Sanger sequencing (Tsingke Biological Technology, Wuhan, China).

### Electroporation transformation and strain selection

Electrocompetent *Z. mobilis* was prepared as described with slight modifications [4]. Briefly, *Z. mobilis* and its derivates were revived from glycerol stock on an RM plate. Then a single colony was picked and inoculated into a 10-mL screw-capped or disposable culture tube at 30 °C, statically overnight. Five milliliters of this overnight culture were then transferred to 500-mL RMG in a capped bottle with an initial OD_600nm_ of 0.025 to 0.03 and cultured at 30 °C until early/mid log phase (OD_600nm_ of 0.4 to 0.6). The culture was chilled on ice for 10 min, and cells were collected at 4 °C, 4000 rpm, 10 min. Cells were resuspended and washed with pre-chilled water once, resuspended, and washed with pre-chilled 10% (v/v) glycerol twice. Finally, cell pellets were resuspended in 10% glycerol at a concentration approximately 1000-fold higher than the starting culture. Competent cells (100 μL) were transferred into 1.5-mL Eppendorf tubes and stored at − 80 °C for later use.

For each electroporation transformation, 50 μL of the competent cells were mixed with 0.5 ~ 5 μg DNA. In 0.1-cm electroporation cuvettes, the cells and plasmid DNA were electroporated (1.6 kV, 25 μF, and 200 ohms) using a Bio-Rad Gene Pulser. The electroporated cells were transferred to 1 mL of mating medium (glucose 50 g/L, yeast extract 10 g/L, tryptone 5 g/L, (NH4)_2_SO_4_ 2.5 g/L, K_2_HPO_4_ 0.2 g/L, 1 mM MgSO_4_), and recovered at 30 °C for 8 to 12 h. Cells were then spread on RM agar plates containing appropriate antibiotics and incubated at 30 °C for 2 to 3 d to isolate single colonies. Since the restriction modification systems in *Z. mobilis* can reduce the transformation efficiency [26], the plasmid was transformed into a methylation-deficient *E. coli* (Trans110) for efficient transformation. The universal primers 15A-fwd/rev were used for colony PCR to check the insert size. Colonies with correct plasmids were cultivated in RMG medium containing appropriate antibiotics for preservation.

### Growth curve analysis using Bioscreen C

Cell growth was monitored by measuring the cell OD values using a Bioscreen C high-throughput growth measurement instrument (Bioscreen C MBR, Helsinki, Finland). Three technical replicates were used for each condition. According to previous work [58], *Z. mobilis* was rather sensitive to 2% butanol and reached only 20% of its growth rate without butanol supplementation. Moreover, *E. coli* cannot tolerate 2% isobutanol (http://2012.igem.org/Team:NCTU_Formosa/Modeling). So, we chose six isobutanol concentrations (0, 4, 8, 12, 16, 20 g/L) to examine the isobutanol toxicity on *Z. mobilis*. Each well of the Bioscreen C plate contained 300 μL bacterium suspension with an initial OD_600nm_ value of ca. 0.05.

### Batch fermentation in shake flasks

Growth curves of *Z. mobilis* ZM4 and its derivates were determined as follows. Strains revived from glycerol stock were inoculated into 5-mL RM media containing appropriate antibiotics and grown overnight without shaking at 30 °C as the seed culture. The seed culture was then transferred into 100-mL shake flasks with 80 mL medium at an initial OD_600nm_ of 0.1. Strains were cultured in shake flakes (30 °C, 100 rpm). Most experiments were performed with medium at 80% of the shake flask volume except for the experiment to investigate the impact of oxygen on isobutanol production using different medium volumes of 20, 50, and 80% of flask volume controlling the amount of dissolved oxygen. For example, the 100-mL shake flasks containing 80 mL RMG (80%) was considered as the least oxygenated anoxic condition, while 100-mL shake flasks containing 20 mL RMG (20%) were regarded as aerobic conditions. Flasks were capped with gas-permeable membrane. Samples from the shake flasks were taken at various time points and the biomass was evaluated by determination of optical density (OD_600nm_) using a spectrometer. Three technical replicates were used for each condition.

### High‑performance liquid chromatography (HPLC) analysis

Samples from the shake flasks were centrifuged at 13,000 rpm for 2 min at 4 °C and then supernatants were filtered through a 0.2-μm syringe filter into HPLC vials. Concentrations of isobutanol, glucose, and ethanol in the supernatant were then determined by an HPLC system (HPLC Prominence, Shimadzu, Japan) equipped with a Bio-Rad Aminex HPX-87H column (Bio-Rad, Hercules, CA, USA) and a refractive index detector (RID). The column temperature was set at 60 °C and 5 mM H_2_SO_4_ solution was used as the mobile phase with a flow rate of 0.5 mL/min.

## Supplementary information

**Additional file 1: Table S1.** The isobutanol yield relative to the maximum theoretical yields (%) for ZMQ3-A2, ZMQ3-A3, and ZMQ3-A4 strains with the induction of tetracycline at concentrations of 0, 0.2 and 1.0 μg/mL (Tc 0, Tc 0.2, and Tc 1.0, respectively) was calculated based on the information of glucose consumed (**Gluc**) and the production of isobutanol (**Iso**) and ethanol (**Eth**) at the time point when most glucose was consumed up and the production of isobutanol and ethanol was the highest (**h**). **Theoretical Isobutanol Titer (g/L)** is the amount of isobutanol that can be produced from all glucose consumed, which was calculated based on the formula: ***Theoretical Isobutanol Titer ***= Glucose Consumed/180.156 (MW of Glucose)*74.122 (MW of Isobutanol). Percentage of theoretical isobutanol maximum yield (Isobutanol yield, %) was then calculated based on the isobutanol produced/Theoretical Isobutanol Titer*100%.

**Additional file 2: Figure S1.** Cell growth, glucose consumption, ethanol and byproduct production of *Z. mobilis* recombinant strain ZMQ3-A4 in flask with different volume of RMG5 medium by the tetracycline induction at different concentrations of 0, 0.2, and 1 μg/mL. Cell growth, glucose (Glu) consumption, and ethanol (Eth) production of ZMQ3-A4 by the tetracycline induction at different concentrations in flask with a volume of medium at 20% **(A)**, 50% **(B)**, and 80% **(C**), as well as the production of byproducts of glycerol, acetoin, and acetate in flask with a volume of medium at 20% **(D)**, 50% **(E)**, and 80% **(F**), respectively. Tc 0, Tc 0.2 and Tc 1 represented the tetracycline concentrations of 0, 0.2, and 1.0 μg/mL that was added into the medium once from the beginning of the experiment, respectively. The results shown are the mean of two technical replicate flasks and the error bars represent standard deviations.

**Additional file 3: Table S2.** Cell growth, glucose consumption, ethanol and byproduct of *Z. mobilis* recombinant strain ZMQ3-A4 for isobutanol production using different concentrations of tetracycline induction in flask with different medium volume 20, 50, 80%, respectively. ND: no determined.

## Data Availability

The authors declare that all the data supporting the findings of this study are available within the paper and its Supplementary Information files or are available from the corresponding author on request.

## Abbreviations

Adh: alcohol dehydrogenase
Als: acetolactate synthase
ED: Entner–Doudoroff
HPLC: high-performance liquid chromatography
HR: homologous recombination
IlvC: ketol-acid reductoisomerase
IlvD: dihydroxy-acid dehydratase
KdcA: 2-ketoacid decarboxylase
Pdc: pyruvate dehydrogenase
RM: rich medium
