# Supplementary material for: Metabolic engineering of Zymomonas mobilis for anaerobic isobutanol production
Source: Biotechnol Biofuels. 2020 Jan 25;13:15. doi: 10.1186/s13068-020-1654-x (PMC6982386; doi:10.1186/s13068-020-1654-x)
Supplement: Supplementary file 1 — Additional file 1: Table S1. The isobutanol yield relative to the maximum theoretical yields (%) for ZMQ3-A2, ZMQ3-A3, and ZMQ3-A4 strains with the induction of tetracycline at concentrations of 0, 0.2 and 1.0 μg/mL (Tc 0, Tc 0.2, and Tc 1.0, respectively) was calculated based on the information of glucose consumed (Gluc) and the production of isobutanol (Iso) and ethanol (Eth) at the time point when most glucose was consumed up and the production of isobutanol and ethanol was the highest (h). Theoretical Isobutanol Titer (g/L) is the amount of isobutanol that can be produced from all glucose consumed, which was calculated based on the formula: Theoretical Isobutanol Titer = Glucose Consumed/180.156 (MW of Glucose)*74.122 (MW of Isobutanol). Percentage of theoretical isobutanol maximum yield (Isobutanol yield, %) was then calculated based on the isobutanol produced/Theoretical Isobutanol Titer*100%. [file 13068_2020_1654_MOESM1_ESM.docx]

**Table S1.** The isobutanol yield relative to the maximum theoretical yields (%) for ZMQ3-A2, ZMQ3-A3, and ZMQ3-A4 strains with the induction of tetracycline at concentrations of 0, 0.2 and 1.0 μg/mL (Tc 0, Tc 0.2, and Tc 1.0 respectively) was calculated based on the information of glucose consumed (**Gluc**) and the production of isobutanol (**Iso**) and ethanol (**Eth**) at the time point when most glucose was consumed up and the production of isobutanol and ethanol was the highest (**h**). **Theoretical Isobutanol Titer (g/L)** is the amount of isobutanol that can be produced from all glucose consumed, which was calculated based on the formula: ***Theoretical Isobutanol Titer****=Glucose Consumed /180.156 (MW of Glucose)*74.122 (MW of Isobutanol).* Percentage of theoretical isobutanol maximum yield (Isobutanol yield, %) was then calculated based on the isobutanol produced/ Theoretical Isobutanol Titer*100%.

| ZMQ3-A2 | Tc 0 | Tc 0.2 | Tc 1.0 |
| --- | --- | --- | --- |
| Time to Maximum Titer (h) | 23.00 | 23.00 | 23.00 |
| Glucose Consumed (g/L) | 46.98 ± 0.36 | 47.04 ± 0.21 | 46.91 ± 0.26 |
| Ethanol Produced (g/L) | 19.97 ± 0.02 | 20.11 ± 0.24 | 18.71 ± 0.17 |
| Isobutanol Produced (g/L) | 0.26 ± 0.01 | 0.32 ± 0.01 | 1.72 ± 0.02 |
| Theoretical Isobutanol Titer (g/L) | 19.33 ± 0.15 | 19.35 ± 0.09 | 19.3 ± 0.11 |
| Isobutanol yield (%) | 1.36 ± 0.06 | 1.65 ± 0.06 | **8.89 ± 0.11** |
|  |  |  |  |
| ZMQ3-A3 | Tc 0 | Tc 0.2 | Tc 1.0 |
| Time to Maximum Titer (h) | 23.00 | 23.00 | 59.00 |
| Glucose Consumed (g/L) | 47.12 ± 0.08 | 46.89 ± 0.32 | 47.15 ± 0.12 |
| Ethanol Produced (g/L) | 20.01 ± 0.05 | 17.49 ± 0.13 | 13.69 ± 0.03 |
| Isobutanol Produced (g/L) | 0.65 ± 0.04 | 3 ± 0.2 | 3.5 ± 0.04 |
| Theoretical Isobutanol Titer (g/L) | 19.38 ± 0.03 | 19.29 ± 0.13 | 19.4 ± 0.05 |
| Isobutanol yield (%) | 3.38 ± 0.19 | 15.56 ± 0.96 | **18.07 ± 0.23** |
|  |  | | |
| ZMQ3-A4 | 0 | 0.2 | 1 |
| Time to Maximum Titer (h) | 23.00 | 31.00 | 59.00 |
| Glucose Consumed (g/L) | 44.74 ± 0.1 | 44.19 ± 1.33 | 41.9 ± 3.19 |
| Ethanol Produced (g/L) | 18.57 ± 0.03 | 13.94 ± 0.55 | 10.93 ± 1 |
| Isobutanol Produced (g/L) | 0.88 ± 0.03 | 3.93 ± 0.18 | 3.93 ± 0.06 |
| Theoretical Isobutanol Titer (g/L) | 18.41 ± 0.04 | 18.18 ± 0.55 | 17.24 ± 1.31 |
| Isobutanol yield (%) | 4.78 ± 0.15 | 21.62 ± 1.38 | **22.88 ± 1.49** |
