# Supplementary material for: Metabolic engineering of Zymomonas mobilis for anaerobic isobutanol production
Source: Biotechnol Biofuels. 2020 Jan 25;13:15. doi: 10.1186/s13068-020-1654-x (PMC6982386; doi:10.1186/s13068-020-1654-x)
Supplement: Supplementary file 2 — Additional file 2: Figure S1. Cell growth, glucose consumption, ethanol and byproduct production of Z. mobilis recombinant strain ZMQ3-A4 in flask with different volume of RMG5 medium by the tetracycline induction at different concentrations of 0, 0.2, and 1 μg/mL. Cell growth, glucose (Glu) consumption, and ethanol (Eth) production of ZMQ3-A4 by the tetracycline induction at different concentrations in flask with a volume of medium at 20% (A), 50% (B), and 80% (C), as well as the production of byproducts of glycerol, acetoin, and acetate in flask with a volume of medium at 20% (D), 50% (E), and 80% (F), respectively. Tc 0, Tc 0.2 and Tc 1 represented the tetracycline concentrations of 0, 0.2, and 1.0 μg/mL that was added into the medium once from the beginning of the experiment, respectively. The results shown are the mean of two technical replicate flasks and the error bars represent standard deviations. [file 13068_2020_1654_MOESM2_ESM.docx]

**Fig. S1. Cell growth, glucose consumption, ethanol and byproduct production of *Z. mobilis* recombinant strain ZMQ3-A4** **in flask with different volume of RMG5 medium by the tetracycline induction at different concentrations of 0, 0.2, and 1 μg/mL.** Cell growth, glucose (Glu) consumption, and ethanol (Eth) production of ZMQ3-A4 by the tetracycline induction at different concentrations in flask with a volume of medium at 20% **(A)**, 50% **(B)**, and 80% **(C**), as well as the production of byproducts of glycerol, acetoin, and acetate in flask with a volume of medium at 20% **(D)**, 50% **(E)**, and 80% **(F**) respectively. Tc 0, Tc 0.2 and Tc 1 represented the tetracycline concentrations of 0, 0.2, and 1.0 μg/mL that was added into the medium once from the beginning of the experiment, respectively. The results shown are the mean of two technical replicate flasks and the error bars represent standard deviations.
